# Small Bowel Follow-Through: Treatment for Small Bowel Obstruction or Delaying the Inevitable?

**DOI:** 10.7759/cureus.50267

**Published:** 2023-12-10

**Authors:** Mohammad Ali, Daniel R Slack, Emery Edmondson, Richard Feinn, Scott H Kurtzman, Zhongqiu J Zhang

**Affiliations:** 1 General Surgery, Waterbury Hospital, Waterbury, USA; 2 Statistics, Frank H. Netter MD School of Medicine, North Haven, USA

**Keywords:** surgery, adhesions, recurrence, sbo, small bowel follow through

## Abstract

Background: Over 400,000 patients are admitted annually for small bowel obstruction (SBO), of which 20-40% require operative intervention, representing more than 2.3 billion dollars in healthcare expenses. Recurrence of SBO increases with a longer duration of follow-up with up to 15-20% recurrence rates within a five-year period. Small bowel follow-through (SBFT) consisting of serial X-rays with oral contrast has been shown to decrease overall length of stay (LOS) in patients with adhesive SBO. The aim of this study is to determine if SBFT administered to patients with SBO decreases 30-day and up to five-year readmission rates secondary to recurrent SBO.

Methods: The institutional review board (IRB) approved a single institution retrospective study from 2010 to 2020 that included a total of 742 patients. These patients were organized into groups of those who received the SBFT <24 hours after admission (n=40), those who received the SBFT >24 hours (n=198), and the third group of patients who did not receive the SBFT (n=658). Readmission rates <30 days, <one year, one to three years, and three to five years were evaluated using analysis of variance. Risk factors such as age groups of <30, 30-50, 50-70, >70 years along with BMI <25, 25-29.9, 30-34.9, 35-39.9, >40, as well as the number of intraabdominal surgeries, gender, and need for operative intervention during the admission were evaluated to assess for any associations with recurrence. Readmission within 30 days and up to five years were compared.

Results: There were no significant differences in recurrence rates between groups with SBFT <24 hours (p=0.338) or SBFT >24 hours (p=0.889) when compared to the no SBFT group. There was nearly a 48% chance of readmission for another episode of an SBO for patients who did not undergo an operative intervention. While patients who underwent operative intervention had around a 29% chance of having a subsequent episode of an SBO. This is consistent with a statistically significant decrease in one-year (p=0.027) recurrences in patients who underwent operative intervention.

Conclusion: There was no significant difference in recurrences with gender, most BMI groups, or in groups who underwent an SBFT. Operative intervention is associated with a statistically significant decrease in recurrence rates of SBO within one year of presentation.

## Introduction

Over 400,000 patients are admitted annually for small bowel obstruction (SBO), of which 20-40% require operative intervention, representing more than 2.3 billion dollars in healthcare expenses [1]. Recurrence of SBO increases with a longer duration of follow-up, with up to 15-20% recurrence rates within a five-year period [2]. Although patients who undergo surgical management do have a decreased likelihood of recurrence, the risk of recurrence remains high. Small bowel follow-through (SBFT) consisting of serial X-rays with oral contrast has been shown to decrease overall length of stay (LOS) in patients with adhesive SBO [3-5]. The SBFT may indirectly decrease recurrence rates for patients who underwent operative intervention, though studies looking at long-term follow-ups after SBFT are limited. The aim of this study is to determine if SBFT administered to patients with SBO decreases 30-day and up to five-year readmission rates secondary to recurrent SBO.

## Materials and methods

This is a single-institution institutional review board (IRB)-approved retrospective study performed at Waterbury Hospital, Waterbury, United States, including a total of 742 patients who had an SBO from 2010 to 2020. The study was performed using either diluted Gastrografin consisting of 90 mL of Gastrografin diluted in 300 mL of water or diluted barium consisting of 176 grams of dry barium diluted in 300 mL of water, but not both. These patients were organized into three groups, SBFT <24 hours after admission (n=40), SBFT >24 hours after admission (n=198), and no SBFT (n=504). A total of 35 out of 40 in the <24 hours group, 170 out of 198 in the >24 hours group, and 450 out of 504 had a nasogastric tube (NGT). All patients without an NGT were secondary to refusal during the admission. Readmission rates ranging from <30 days to up to five years were compared. In the SBFT <24 hours group, (50%) received diluted Gastrografin while the remaining 20 patients (50%) received diluted barium. In the SBFT >24 hours group, 88 patients (64%) received diluted Gastrografin and 110 patients (44%) received diluted barium. A total of 108 patients (45%) received diluted Gastrografin while 130 patients (55%) received diluted barium in the patients who received an SBFT.

Inclusion criteria were SBO secondary to adhesions from prior surgery, obstipation, and CT scan findings confirming a moderate to high-grade obstruction. Exclusion criteria were patients who had an SBO secondary to inflammatory bowel disease, foreign body, malignancy, perforation, or hemodynamic instability requiring emergent operative intervention and SBO without any prior surgical history. Primary outcomes that were evaluated included readmission rates <30 days, one year, one to three years, and three to five years; they were evaluated using analysis of variance. Secondary associations with recurrence of an SBO were assessed using variables such as age, BMI, number of intra-abdominal surgeries, gender, and need for operative intervention during the admission.

An analysis of variance using the Brown-Forsythe robust test was used to compare the three groups for continuous outcomes. A Pearson Chi-square, Fisher’s Exact Test, and Linear by Linear association were used to assess for SBO recurrences with any associations with age, BMI, number of intra-abdominal surgeries, gender, and need for operative intervention during the admission.

## Results

The total of 742 patients with an SBO included 238 patients who received an SBFT and 504 patients who did not receive an SBFT. Of the 238 patients who received an SBFT, 40 received the study <24 hours after admission while 198 received the study >24 hours after admission. In the SBFT group, there were a total of 99 readmissions consisting of nearly 42% of the 238 patients in this study group. A total of 20 readmissions <30 days (8.4%), 34 within <one year (14.2%), 30 patients within one to three years (12.6%), and 15 patients within three to five years (6.3%).

In the subgroup of patients that received the SBFT <24 hours, there were a total of 11 readmitted patients comprising around 26% of the 40 patients in this group (Table 1). Only one patient was readmitted <30 days (2.5%), four patients within <one year (10%), two patients within one to three years (5%), and four patients within three to five years (10%). Out of the 504 patients who did not undergo an SBFT study, there were a total of 264 readmissions consisting of a nearly 52% recurrence rate. A total of 44 patients had readmission <30 days (8.7%), 98 patients within <one year (19.4%), 87 patients within one to three years (17.2%), and 35 patients within three to five years (6.9%). An analysis of variance with the Brown-Forsythe robust test was used to evaluate for any statistical significance for readmission after the administration of an SBFT or operative intervention.

**Table 1:** Recurrence of SBO and associated risk factors OR: Operating room; SBFT: Small bowel follow-through; hrs: hours; yrs: years

Recurrence timing	OR intervention	SBFT timing (hrs)	Previous surgeries	BMI kg/m²	Age (yrs)	Gender
<30 days	Yes (p=0.79) No (p=0.79)	<24 hrs (p=0.889) >24 hrs (p=0.889) No SBFT (p=0.889)	1-2 (p=0.125) 2-5 (p=0.125) >5 (p=0.125)	<25 (p=0.154) 25-29.9 (p=0.154) 30-34.9 (p=0.154) 35-39.9 (p=0.154) >40 (p=0.154)	<30 (p=3.938) 30-50 (p=3.938) 50-70 (p=3.938) >70 (p=3.938)	Male (p=0.441) Female (p=0.441)
<1 year	Yes (p=0.027) No (p=0.641)	<24 hrs (p=0.338) >24 hrs (p=0.889) No SBFT (p=0.664)	1-2 (p=0.125) 2-5 (p=0.125) >5 (p=0.125)	<25 (p=0.003) 25-29.9 (p=0.154) 30-34.9 (p=0.154) 35-39.9 (p=0.154) >40 (p=0.154)	<30 (p=0.478) 30-50 (p=0.478) 50-70 (p=0.478) >70 (p=0.478)	Male (p=0.507) Female (p=0.507)
1-3 years	Yes (p=0.037) No (p=0.262)	<24 hrs (p=0.107) >24 hrs (p=0.107) No SBFT (p=0.107)	1-2 (p=0.125) 2-5 (p=0.125) >5 (p=0.125)	<25 (p=0.04) 25-29.9 (p=0.217) 30-34.9 (p=0.217) 35-39.9 (p=0.217) >40 (p=0.217)	<30 (p=1.360) 30-50 (p=1.360) 50-70 (p=1.360) >70 (p=1.360)	Male (p=0.88) Female (p=0.88)
3-5 years	Yes (p=0.02) No (p=0.887)	<24 hrs (p=0.490) >24 hrs (p=0.490) No SBFT (p=0.490)	1-2 (p=0.125) 2-5 (p=0.125) >5 (p=0.125)	<25 (p=0.386) 25-29.9 (p=0.386) 30-34.9 (p=0.386) 35-39.9 (p=0.386) >40 (p=0.386)	<30 (p=7.398) 30-50 (p=7.398) 50-70 (p=7.398) >70 (p=7.398)	Male (p=0.66) Female (p=0.66)

Out of the patients who did not undergo operative intervention, there was nearly a 48% chance of readmission for another episode of an SBO. While patients who underwent operative intervention had around a 29% chance of having a subsequent episode of an SBO. This is consistent with a statistically significant decrease in one-year (p=0.027), one- to three-year (p=0.0037), and three- to five-year (p=0.02) recurrences in patients who underwent operative intervention. There were no significant differences in recurrence rates between groups with SBFT <24 hrs (p=0.338) or SBFT >24 hrs (p=0.889) when compared to the no SBFT group. No statistical difference between age (p=0.268), number of intra-abdominal surgeries (p=0.125), gender (p=0.507), or BMI groups >25 (p=0.154). There was a statistically significant increase rate of one-year (p=0.003) and one- to three-year (p=0.04) recurrences of SBO associated with BMI <25.

## Discussion

Non-operative management has become the mainstay for stable patients with adhesive SBO. Previous studies have demonstrated that the use of oral contrast including water-soluble contrast and barium can be diagnostic as well as therapeutic for an SBO [1]. While this can decrease time to surgery, LOS, and hospital costs, it is unclear if it has any effect on recurrences. Unfortunately, recurrence rates for SBO are quite high and increase with each admission [3], raising the question if this can be reduced. Multiple studies have demonstrated that surgical intervention decreases re-admissions for SBO [4]; however, to our knowledge, no other interventions have confidently demonstrated a reduction.

Our retrospective study of 742 patients with a follow-up period ranging from 30 days to five years demonstrated that SBFT did not have a significant impact on the recurrence of SBO. Interestingly, the SBFT within the 24-hour group did show fewer recurrence rates; however, these were not enough to reach statistical significance. This may be related to the fact that SBFT tends to increase operative intervention rates, which does demonstrate a significant reduction in recurrence. Our study demonstrated that patients who underwent operative intervention had significantly lower recurrences similar to prior studies’ results.

We found no difference in recurrence in patients based on age or gender. Previous studies have suggested that those patients who undergo SBFT tend to undergo operative intervention more often and sooner [5]. This is critical in the management of SBO because patients who undergo operative intervention earlier during their admission consisting of less than four days of hospital stay tend to have a decreased overall morbidity and mortality rates [4-6]. Unfortunately, it seems this effect of increased likelihood for surgical intervention did not have enough impact to reduce recurrences in SBFT patients.

Due to the retrospective nature of the study, direct correlations cannot be drawn and the potential for selection bias remains. The limitations of this study include the non-randomized nature of the study performed in a single community hospital setting. Furthermore, patients who were unable to undergo surgery secondary to comorbidities including chronic obstructive pulmonary disease, coronary artery disease, diabetes, and decreased functional status were not stratified. Although we had a large sample size, it is likely that some SBO recurrences presented at outside hospitals and were not included in our data. While we attempted to include comorbidities and factors we felt would likely have an impact on recurrences, there is a potential for confounders that were not assessed. 

The use of SBFT has a benefit to patients admitted with an SBO acutely but does not seem to affect the recurrence rates for better or for worse. SBFT has been shown in prior studies to increase the likelihood of a patient undergoing operative intervention which ours and other studies have shown to reduce recurrence [7-8]. The limitations of this paper include this potential confounder of SBFT indirectly decreasing recurrences in those patients who underwent surgery, and it being a single institution retrospective study. The use of SBFT should be continued with perhaps a lower threshold by surgeons to take a patient for operative intervention, especially if this is not their first SBO given the high recurrence rate. Naturally, the benefits of potentially reducing SBO recurrence should be weighed against the risk of operative intervention in potentially hostile abdomens or poor surgical candidates with multiple comorbidities. Further studies are needed to determine other risk factors in the recurrence of SBO.

## Conclusions

There was no significant difference in recurrences with gender, most BMI groups, or groups who underwent an SBFT in our study. Operative intervention is associated with a statistically significant decrease in recurrence rates of SBO within one year of presentation. Further large-scale randomized studies can be considered to further delineate the utility of SBFT and the outcomes of SBO recurrences.
